# Variability in body weight and the risk of cardiovascular complications in type 2 diabetes: results from the Swedish National Diabetes Register

**DOI:** 10.1186/s12933-021-01360-0

**Published:** 2021-08-26

**Authors:** Antonio Ceriello, Giuseppe Lucisano, Francesco Prattichizzo, Björn Eliasson, Stefan Franzén, Ann-Marie Svensson, Antonio Nicolucci

**Affiliations:** 1 IRCCS MultiMedica, Via Gaudenzio Fantoli, 16/15, 20138 Milan, Italy; 2 CORESEARCH - Center for Outcomes Research and Clinical Epidemiology, Pescara, Italy; 3 Institute of Medicine, University of Gothenburg, Gothenburg, Sweden; 4 Health Metrics, Department of Public Health and Community Medicine, Sahlgrenska Academy, University of Gothenburg, Gothenburg, Sweden; 5 Center for Registries, Västra Götaland, Gothenburg, Sweden

**Keywords:** Cardiovascular complications, Diabetes, Swedish National Diabetes Register, Weight variability

## Abstract

**Background:**

There is a high incidence of cardiovascular disease in diabetes. Weight variability has been reported as independent risk factor for cardiovascular disease in the general population and preliminarily also in people with type 2 diabetes.

**Methods:**

Using data from the Swedish National Diabetes Register the possible link between visit-to-visit body weight variability and the risk of cardiovascular complications among people with type 2 diabetes and without prevalent cardiovascular diseases at baseline has been evaluated. Overall, 100,576 people with type 2 diabetes, with at least five measurements of body weight taken over three consecutive years, were included. Variability was expressed as quartiles of the standard deviation of the measures during the three years. The primary composite outcome included non-fatal myocardial infarction, non-fatal stroke, and all-cause mortality and was assessed during five years following the first 3 years of exposure to weight variability.

**Results:**

After adjusting for known cardiovascular risk factors, the risk of the primary composite outcome significantly increased with increasing body weight variability [upper quartile HR = 1.45; 95% confidence interval 1.39–1.52]. Furthermore, elevated body weight variability was associated with almost all the other cardiovascular complications considered (non-fatal myocardial infarction, non-fatal stroke, all-cause mortality, peripheral arterial disease, peripheral vascular angioplasty, hospitalization for heart failure, foot ulcer, and all-cause mortality).

**Conclusions:**

High body weight variability predicts the development of cardiovascular complications in type 2 diabetes. These data suggest that any strategy to reduce the body weight in these subjects should be aimed at maintaining the reduction in the long-term, avoiding oscillations.

**Supplementary Information:**

The online version contains supplementary material available at 10.1186/s12933-021-01360-0.

## Introduction

It has been reported that over-time body weight variability (BWV) may increase the risk for cardiovascular diseases (CVD) in the general population [1–3] or in people with an established CVD [4]. People with diabetes show an increase of the incidence of CVD [5]. BWV as cardiovascular risk factor has also been reported in type 2 diabetes (T2D), as shown in post-hoc analyses of clinical trials [6–8]. These data were recently supported by a large, longitudinal, real-world study with Asian patients, which showed that BWV was associated with increased risks of MI, stroke, and all-cause mortality in patients with T2D [9]. However, to our knowledge, similar findings in Caucasian patients are missing.

The present study evaluated the possible link between visit-to-visit BWV, the risk of CVD among people with T2D and without prevalent cardiovascular diseases at baseline, using data of 100,576 patients from the Swedish National Diabetes Register (NDR) [10].

## Methods

### Population

The database consulted derives from the NDR. The NDR, initiated in 1996, has been described previously [10]. This registry includes information on risk factors, complications of diabetes, and medications for patients 18 years of age or older. All patients have consented to being reported in NDR, while no individual consent is required to be included in this study according to Swedish law. The regional ethical review board approved this study protocol. We used nationwide data sources in Sweden, including population registers and Statistics Denmark/Statistics Sweden (vital status, demographics, socioeconomic variables), patient registers (comorbidities, outcomes), prescription registers (study drugs, co-medications), cause of death registers (outcomes), the NDR. The data sources are described in detail in the Additional file 1: Table S1.

Data of patients with T2D collected in successive visits in the NDR between January 1st 2000 and September 25th 2019 were considered for this study. Information collected included gender, age, smoking, diabetes duration, measurements of: HbA1c, body weight, blood pressure, serum creatinine, urinary albumin excretion, total-cholesterol, low-density lipoprotein cholesterol (LDL), high-density lipoprotein cholesterol (HDL), and triglycerides. Information on antihyperglycemic treatment (diet, oral agents, insulin, oral agents + insulin), antihypertensive treatment (yes vs. no), lipid-lowering treatment (yes vs. no), and aspirin (yes vs. no) was also collected.

The estimated glomerular filtration rate (eGFR) was determined for each patient by using the “Modification of Diet in Renal Disease” equation. Albuminuria was categorized as normal, microalbuminuria, and macroalbuminuria. The presence of diabetes complications (retinopathy, cardio-cerebrovascular, heart failure, peripheral arterial disease, minor and major amputations) was also registered, using the “International Classification of Diseases, 9th Revision and 10th Revision”. The specific codes are listed in Additional file 1: Table S1 in the Appendix.

The primary outcome was represented by a composite of first occurrence of non-fatal myocardial infarction, non-fatal stroke, and all-cause mortality.

The following secondary outcomes were considered: non-fatal-myocardial infarction, non-fatal stroke, all-cause mortality, coronary artery bypass graft surgery (CABG), percutaneous coronary intervention (PCI), peripheral arterial disease, peripheral vascular angioplasty, hospitalization for heart failure, foot ulcer. An expanded composite outcome including non-fatal myocardial infarction, non-fatal stroke, CABG, PCI, peripheral revascularization procedures, and all-cause mortality was also considered.

**Fig. 1:**
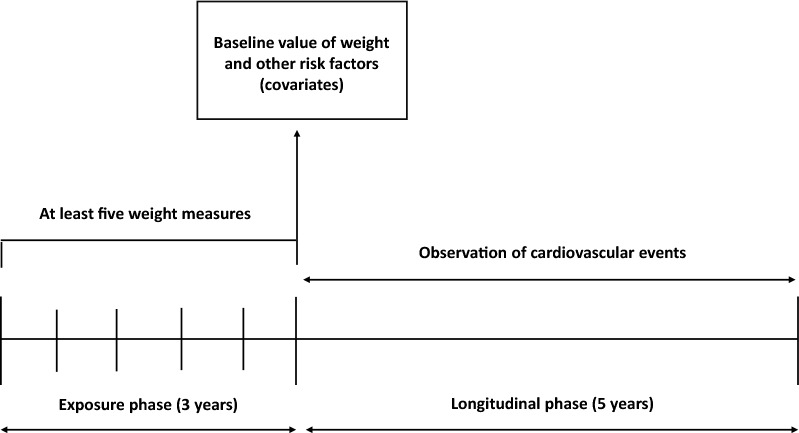
Schematic representation of the experimental design of the study

Within the database, we identified all patients with at least 5 measurements of body weight taken over a period of three consecutive years from the first visit. Starting from the end of the third year of observation (exposure phase), those patients with no history of major cardiovascular events were followed up to the latest available visit (longitudinal phase) (Fig. 1). Patients with 5 measures of body weight diluted in a period longer than 3 years and patients experiencing an event during the exposure phase were excluded.

### Statistical analysis

Descriptive data are summarized as median and interquartile range for continuous variables and as percentages for categorical variables. The relation between BWV and the risk of outcomes was evaluated with the use of BWV as a categorical variable. BWV was calculated as the standard deviation, i.e. the square root of the variance, of the body weight measures available during the three years preceding the longitudinal phase of the study. Standard deviation was chosen since it is the most used metric to assess the variability of risk factors [11]. A minimum of five measures was considered, in order to have a robust estimate of variability. Patients were thus grouped according to the quartiles for BWV to ensure 4 groups with different variability but equal size.

The association between BWV and risk of developing the outcomes of interest was investigated through multivariate Cox proportional-hazards regression analyses. Each Cox model also included the following baseline covariates: age, gender, duration of diabetes, body weight, smoking, values of HbA1c, systolic and diastolic blood pressure, total cholesterol, HDL, LDL, triglycerides, albuminuria, eGFR, retinopathy, treatment for diabetes, hypertension, dyslipidemia, and aspirin use. In all Cox models, patients were censored at the last visit. Results are expressed as hazard ratios (HRs) with their 95% confidence interval (95% CI). The rate of outcomes was evaluated for each of the quartiles of BWV, with the lowest quartile used as the reference category. A p-value for trend was estimated to assess of the presence of a linear association between increasing BWV and increasing risk of the outcomes. The same analysis was also repeated separately by gender. To manage missing data relative to covariates, a category of missing data was added for each covariate in the multivariate analysis. However, such numbers were negligible (data not shown).

Tests were 2-sided, and a p value < 0.05 was considered statistically significant. Statistical analyses were performed with SAS software, version 9.4 (SAS Institute Inc. North Carolina, USA).

## Results

In total, 100,576 patients without established CVD were available for the calculation of BWV. Characteristics of patients by BWV quartiles are reported in Table 1. Patients in the upper quartile of BWV were younger, had shorter diabetes duration, and a higher prevalence of smokers. Weight increased with increasing levels of BWV, while an opposite trend was documented for HbA1c.

**Table 1:** Characteristics of the study population by quartiles of body weight variability

Characteristic	Quartiles of body weight variability				p-value
	**I**	**II**	**III**	**IV**	
No. of patients	24,602	25,315	25,453	25,206	
Mean interval between BW measurements in years (std)	0.5 (0.08)	0.6 (0.09)	0.6 (0.08)	0.5 (0.07)	NS
Weight SD	0.9 (0.6–1.1)	1.7 (1.5–1.9)	2.6 (2.3–2.9)	4.5 (3.8–6.1)	< 0.0001
Gender (% males)	52.9	56.1	57.5	56,0	< 0.0001
Age (years)	66.0 (58.0–73.0)	65.0 (57.0–72.0)	64.0 (55.0–71.0)	62.0 (53.0–69.0)	< 0.0001
Smoking	14.0	14.6	15.9	18.2	< 0.0001
BMI	28.1 (25.3–31.4)	29.0 (26.1–32.4)	29.6 (26.6–33.5)	30.7 (27.0–35.1)	< 0.0001
Body weight	81.3 (71.6–92.0)	85.0 (74.5–96.0)	88.0 (77.0–100.0)	91.1 (78.8–106.0)	< 0.0001
Duration of diabetes					< 0.0001
≤ 2 years	14.8	15.4	16.9	18.8	
2.1–5 years	48.2	53.7	57.5	58.5	
5.1–10 years	18.5	15.8	13.5	12.0	
> 10 years	18.5	15.1	12.1	10.6	
HbA1c (mmol/mol)	51.0 (45.0–58.0)	50.0 (45.0–58.0)	49.0 (44.0–57.0)	47.0 (42.0–56.0)	< 0.0001
Systolic blood pressure (mmHg)	135 (125–144)	135 (125–142)	134 (125–140)	132 (124–140)	< 0.0001
Diastolic blood pressure (mmHg)	80 (70–82)	80 (70–84)	80 (70–84)	80 (71–85)	< 0.0001
Total cholesterol (mmol/l)	4.7 (4.1–5.4)	4.6 (4.0–5.4)	4.6 (4.0–5.4)	4.6 (4.0–5.4)	< 0.0001
HDL cholesterol (mmol/l)	1.2 (1.0–1.5)	1.2 (1.0–1.5)	1.2 (1.0–1.5)	1.2 (1.0–1.5)	< 0.0001
LDL cholesterol (mmol/l)	2.6 (2.1–3.3)	2.6 (2.0–3.3)	2.6 (2.0–3.2)	2.6 (2.0–3.3)	< 0.0001
Triglycerides (mmol/l)	1.5 (1.1–2.1)	1.6 (1.1–2.2)	1.6 (1.1–2.2)	1.5 (1.1–2.2)	< 0.0001
Albuminuria					< 0.0001
No albuminuria	75.9	77.2	77.2	76.4	
Microalbuminuria	13.5	14.0	14.2	14.3	
Macroalbuminuria	3.4	3.3	3.4	4.0	
Not available	7.2	5.5	5.4	4.0	
eGFR (ml/min/1.73m^2^)	82.7 (69.2–97.8)	83.5 (70.0–98.3)	84.8 (70.8–99.8)	86.4 (72.1–101.6)	< 0.0001
Diabetes retinopathy	16.4%	16.7%	15.7%	16.1%	< 0.0001
Diabetes treatment					< 0.0001
Lifestyle only	15.9	15.0	14.5	18.0	
Oral agents	64.3	65.6	65.9	60.8	
Insulin	7.3	6.8	7.5	8.6	
Insulin + oral agents	12.5	12.6	12.1	12.6	
Antihypertensive medication	70.1	69.9	70.0	68.3	< 0.0001
Statin medication	59.0	59.6	59.0	54.7	< 0.0001
Aspirin	23.3	22.3	20.3	17.5	< 0.0001

The median follow-up time of the longitudinal phase was 4.4 years (range 2.1–6.7).

### Development of complications

**Fig. 2:**
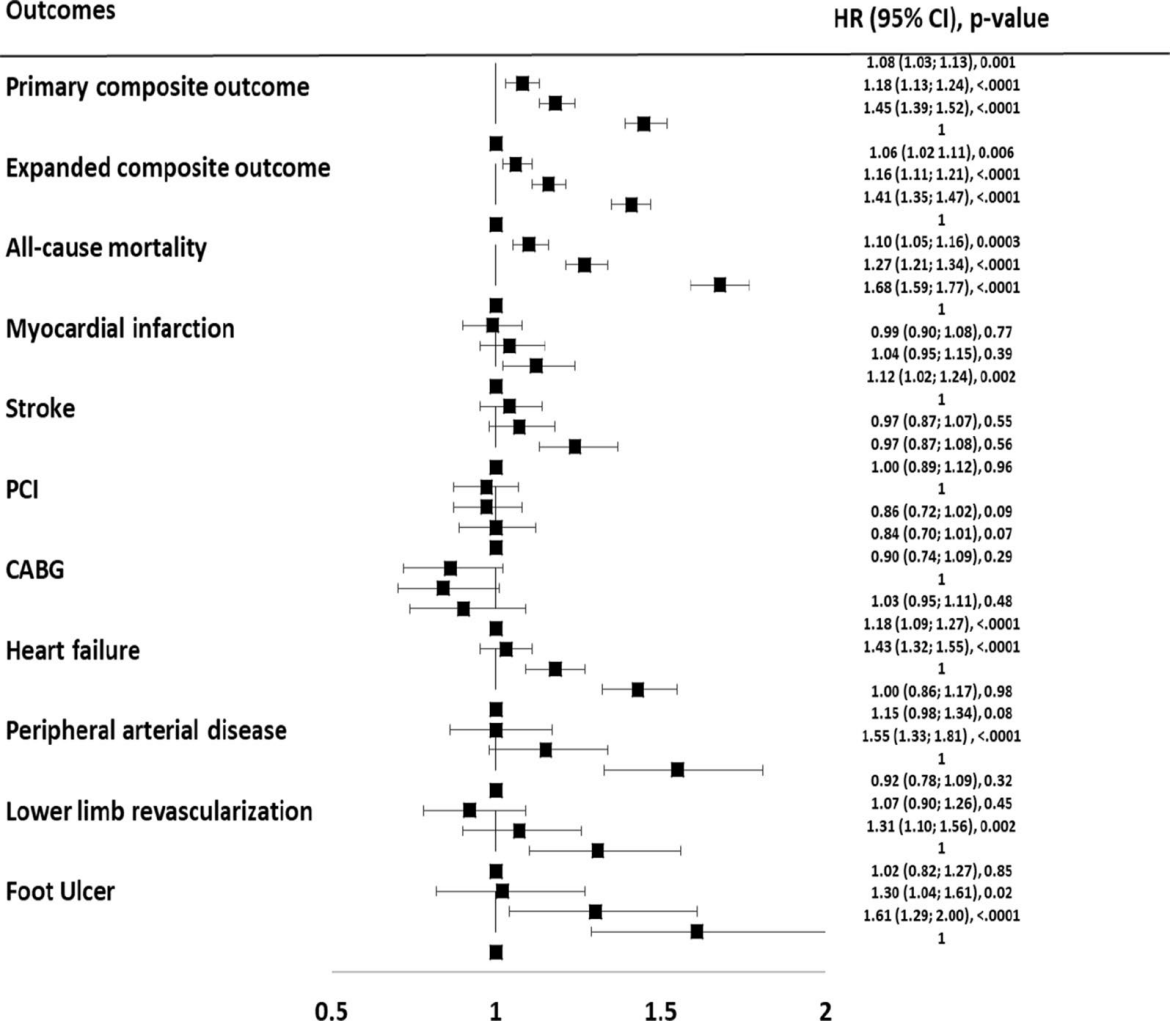
Forest plot summarizing the adjusted hazard ratio (HR) along with the 95% Confidence Interval (CI) in quartile 2, quartile 3, and quartile 4 compared to quartile 1 for all the outcomes assessed

The association between the measure of intra-individual BWV and the development of the different outcomes, adjusted for all the other factors described above, is reported in Fig. 2, while Additional file 1: Table S2 of the Appendix reports the number and the event-rate of each outcome in the four groups considered.

### *Primary outcome*

Figure 2 shows the primary composite outcome represented by non-fatal myocardial infarction, non-fatal stroke, and all-cause mortality significantly increased with increasing BWV. Compared to the lowest quartile of BWV, the risk of the primary outcome increased by 8% in the second quartile (HR = 1.08; 95% CI 1.03–1.13), by 18% in the third quartile (HR = 1.18; 95% CI 1.13–1.24), and by 45% in the upper quartile (HR = 1.45; 95% CI 1.39–1.52).

### Secondary outcomes

In the secondary outcomes analyses a linear increase in the risk of event associated with increasing BWV was documented for most of the outcomes considered. In particular, compared to patients in the lowest quartile of BWV, those in the upper quartile had 68% increased risk of all-cause mortality (HR = 1.68; 95% CI 1.59–1.77), 12% increased risk of myocardial infarction (HR = 1.12; 95% CI 1.02–1.24), 24% increased risk of stroke (HR = 1.24; 95% CI 1.13–1.37), 43% higher risk of heart failure (HR = 1.43; 95% CI 1.32–1.55), 55% higher risk of peripheral arterial disease (HR = 1.55; 95% CI 1.33–1.81), and 61% higher risk of foot ulcers (HR = 1.61; 95% CI 1.29–2.00). The risk of the expanded composite outcome increased across quartiles of weight variability, with an excess risk of 41% for the upper quartile compared to the lowest quartile (HR = 1.41; 95% CI 1.35–1.47) (Fig. 2). All the outcomes tested, with the exception of myocardial infarction, percutaneous coronary intervention, and coronary artery bypass graft surgery, showed a significant p for trend (Table 2), thus suggesting a progressively increasing risk along with growing quartiles of BWV.

**Table 2:** Hazard ratios for each of the assessed outcome considering the entire cohort and separately males/females, along with the p for trend among quartiles for each outcome and the relative p for interaction for the sex-based subgroup analysis

MODEL	Label	Overall			Male			Female			
		**HRCI**	**ProbChi** **Sq**	**p for trend**	**HRCI**	**ProbChi** **Sq**	**p for trend**	**HRCI**	**ProbChi** **Sq**	**p for trend**	**p for interaction**
Primary Composite Outcome	vikt_STD_Q2	1.08 (1.03–1.12)	0.0012	< .0001	1.11 (1.05–1.18)	0.0005	< .0001	1.03 (0.97–1.11)	0.3152	< .0001	0.539
	vikt_STD_Q3	1.18 (1.13–1.24)	< .0001		1.20 (1.13–1.28)	< .0001		1.16 (1.08–1.24)	< .0001		
	vikt_STD_Q4	1.45 (1.39–1.52)	< .0001		1.46 (1.37–1.55)	< .0001		1.46 (1.36–1.56)	< .0001		
Expanded Composite Outcome	vikt_STD_Q2	1.06 (1.02–1.11)	0.0057	< .0001	1.08 (1.02–1.15)	0.0050	< .0001	1.03 (0.97–1.10)	0.3296	< .0001	0.823
	vikt_STD_Q3	1.16 (1.11–1.21)	< .0001		1.16 (1.10–1.23)	< .0001		1.15 (1.08–1.23)	< .0001		
	vikt_STD_Q4	1.41 (1.35–1.47)	< .0001		1.39 (1.31–1.48)	< .0001		1.43 (1.34–1.53)	< .0001		
All cause mortality	vikt_STD_Q2	1.10 (1.05–1.16)	0.0003	< .0001	1.12 (1.05–1.21)	0.0015	< .0001	1.08 (1.00–1.17)	0.0393	< .0001	0.527
	vikt_STD_Q3	1.27 (1.21–1.34)	< .0001		1.31 (1.21–1.41)	< .0001		1.23 (1.14–1.33)	< .0001		
	vikt_STD_Q4	1.68 (1.59–1.77)	< .0001		1.72 (1.60–1.85)	< .0001		1.63 (1.51–1.76)	< .0001		
MI	vikt_STD_Q2	0.99 (0.90–1.08)	0.7542	0.06	1.03 (0.92–1.16)	0.5893	0.81	0.91 (0.79–1.06)	0.2412	0.01	0.085
	vikt_STD_Q3	1.04 (0.65–1.15)	0.3902		1.04 (0.92–1.18)	0.5205		1.04 (0.89–1.22)	0.5924		
	vikt_STD_Q4	1.12 (1.02–1.24)	0.0225		1.07 (0.94–1.22)	0.3382		1.23 (1.05–1.44)	0.0109		
Stroke	vikt_STD_Q2	1.04 (0.95–1.14)	0.3497	0.0002	1.11 (0.98–1.25)	0.0879	0.003	0.97 (0.85–1.11)	0.6472	0.03	0.366
	vikt_STD_Q3	1.07 (0.98–1.18)	0.1344		1.09 (0.96–1.23)	0.2022		1.06 (0.93–1.22)	0.3750		
	vikt_STD_Q4	1.24 (1.13–1.37)	< .0001		1.28 (1.12–1.47)	0.0002		1.20 (1.04–1.38)	0.0151		
PCI	vikt_STD_Q2	0.97 (0.87–1.07)	0.5287	0.86	0.98 (0.87–1.11)	0.7819	0.69	0.92 (0.76–1.12)	0.4182	0.19	0.447
	vikt_STD_Q3	0.97 (0.87–1.08)	0.5657		0.94 (0.81–1.07)	0.3136		1.04 (0.86–1.27)	0.6750		
	vikt_STD_Q4	1.00 (0.89–1.12)	0.9597		0.94 (0.81–1.08)	0.3517		1.16 (0.95–1.42)	0.1394		
CABG	vikt_STD_Q2	0.86 (0.72–1.02)	0.0858	0.23	0.84 (0.69–1.03)	0.0939	0.27	0.92 (0.64–1.33)	0.6639	0.90	0.701
	vikt_STD_Q3	0.84 (0.70–1.01)	0.0707		0.84 (0.68–1.03)	0.0892		0.86 (0.58–1.29)	0.4640		
	vikt_STD_Q4	0.90 (0.74–1.09)	0.2915		0.88 (0.71–1.10)	0.2711		0.95 (0.63–1.44)	0.8197		
HF	vikt_STD_Q2	1.03 (0.95–1.11)	0.4831	< .0001	1.07 (0.96–1.19)	0.2308	< .0001	0.99 (0.89–1.10)	0.8338	< .0001	0.828
	vikt_STD_Q3	1.18 (1.09–1.27)	< .0001		1.26 (1.13–1.39)	< .0001		1.09 (0.98–1.22)	0.1262		
	vikt_STD_Q4	1.43 (1.32–1.55)	< .0001		1.48 (1.32–1.65)	< .0001		1.39 (1.24–1.55)	< .0001		
PAD	vikt_STD_Q2	1.00 (0.86–1.17)	0.9761	< .0001	1.03 (0.85–1.26)	0.7363	0.002	0.95 (0.75–1.21)	0.6702	< .0001	0.598
	vikt_STD_Q3	1.15 (0.98–1.34)	0.0833		1.10 (0.90–1.35)	0.3560		1.20 (0.94–1.53)	0.1342		
	vikt_STD_Q4	1.55 (1.33–1.81)	< .0001		1.43 (1.17–1.75)	0.0006		1.69 (1.34–2.14)	< .0001		
Lower limb revascularization	vikt_STD_Q2	0.92 (0.78–1.09)	0.3244	0.001	0.96 (0.77–1.20)	0.7338	0.05	0.87 (0.68–1.12)	0.2895	0.04	0.801
	vikt_STD_Q3	1.07 (0.90–1.26)	0.4522		1.13 (0.90–1.41)	0.3087		1.00 (0.77–1.29)	0.9779		
	vikt_STD_Q4	1.31 (1.10–1.56)	0.0022		1.32 (1.04–1.67)	0.0230		1.30 (1.01–1.67)	0.0447		
Foot Ulcer	vikt_STD_Q2	1.02 (0.82–1.27)	0.8462	< .0001	1.01 (0.75–1.37)	0.9373	0.05	1.01 (0.73–1.40)	0.9364	0.001	0.239
	vikt_STD_Q3	1.30 (1.04–1.61)	0.0194		1.25 (0.93–1.69)	0.1380		1.31 (0.95–1.79)	0.0989		
	vikt_STD_Q4	1.61 (129–2.00)	< .0001		1.45 (1.07–1.97)	0.0171		1.73 (1.27–2.36)	0.0006		

### Subgroup analysis

To assess if sex influenced the observed results, we repeated the analysis considering separately men and women. We obtained comparable results for both sexes, with no evident interaction among these subgroups (Table 2).

## Discussion

We used nationwide register data from NDR to assess the possible impact of BWV on cardiovascular complications development in T2D. The results presented here show that high BWV is predictive of almost all of cardiovascular complications in T2D. Our study is the first reporting data on the effects of BWV on cardiovascular complications in Caucasian subjects with T2D, who were free of such complications at the entry, in the real life, in a huge number of subjects and with a long follow-up. Our data clearly shows that high BWV is strongly correlated to a higher risk for cardiovascular complications in T2D, even when corrected for the major possible confounding factors, an effect equally observed in men and women.

Several years ago, the first evidence that BWV could be related to high risk of CVD in the general population emerged from the Framingham Study [1]. In the context of diabetes, data from three clinical trials were pooled and used to evaluate the impact of BWV in 6,408 patients with T2D on the development of macrovascular endpoints, using a composite of coronary heart disease death, myocardial infarction, resuscitated cardiac arrest, coronary revascularization, and unstable or new-onset angina as the primary endpoint [6]. When used as a time-dependent covariate, BWV, measured as average successive variability, was linearly and independently associated with an increased risk of any coronary event, major coronary event, any cardiovascular event, and death [6]. In particular, when comparing the highest with the lowest quintile of BWV, the increased risk for any component of the composite outcome was substantially higher [6]. These results suggest that among subjects with T2D, fluctuation in body weight is associated with higher mortality and a higher rate of cardiovascular events, independent of traditional cardiovascular risk factors [6].

The Action to Control Cardiovascular Risk in Diabetes (ACCORD) trial participants' weight was documented annually during the trial [7]. Out of the 10,251 ACCORD participants, 911(8.9%) had normal weight, 2,985 (29.1%) were overweight, and 6,355(62%) were obese. During a mean of 3.5 years of follow-up, BWV was associated with the primary outcome MACE, but also with heart failure, death, and microvascular events, an observation independent of cardiovascular risk factors and BMI [7]. Our data also shows a correlation between BWV and total mortality, independently of the age. A previous study, in a smaller number of subjects (1, 319), reported such correlation but only in the elderlies [12]. The mechanism through which BWV may increase the risk for cardiovascular complications in patients with or without diabetes remains to be elucidated [13]. BWV is not associated with a worsening of cardiovascular risk factors, suggesting that the oscillation of canonical risk factors may not mediate the deleterious effect of BWV on the cardiovascular system [14]. Weight cycling is associated with increased food efficiency and increased caloric consumption, which can lead to adipose hypertrophy, that can generate inflammation and oxidative stress [13, 15, 16]. Studies in humans and in animals show that BWV induces low-grade inflammation and oxidative stress, [13, 15–17] two conditions that can favour the development of insulin resistance, [16, 17] which, in turn, can lead to cardiovascular complications [18]. On the other hand, both low-grade inflammation and oxidative stress can directly promote the development of cardiovascular complications in diabetes [19–21]. Transcriptomic studies in obese patients subjected to weight cycling showed that weight gain after weight loss promote the expression of a number of genes involved in the activation of pathways related to the formation of fibrin clot, cardiomyopathy, and cell surface interaction at vascular wall, three key phenomena in the development of CVD [22]. Of note, these pathways were not affected by weight loss, but only showed modulation after weight re-gain [22], a finding confirmed in another study and especially relevant for inflammatory and hypertrophic pathways [23]. In addition, weight loss only marginally affected a number of altered transcriptomic signatures, suggesting that weight gain induces enduring alterations [22].

Our finding might raise relevant issues for diabetes management. Body weight reduction remains a key strategy for risk reduction [24] since it is accompanied by an improvement of cardiovascular risk factors in T2D [25]. The real benefit of this intervention remains, however, unclear. In the randomized Look Action for Health in Diabetes [Look AHEAD] trial, an intensive lifestyle intervention focusing on weight loss did not reduce the rate of cardiovascular events in overweight or obese adults with T2D [26]. However, a post-hoc analysis of the study suggested an association between the magnitude of weight loss and incidence of cardiovascular disease in people with T2D [27]. On the other hand, weight reduction is followed very often by regaining weight, and this “yo–yo” effect is frequently present in the long-life management of diabetes [28].

Patient’s weight is among the clinical features to be considered when additional drugs are prescribed to optimize glycemic control in patients with diabetes [24]. Insulin, glitazons, and sulphonylureas are known to promote weight gain, while metformin and the more recently introduced sodium-glucose transporters 2 inhibitors and glucagon-like receptor 1 agonists are held to produce a durable, albeit small, weight loss [29]. The findings of our study might support the use of the latter drugs, already prioritized in patients with cardiovascular diseases, in obese patients with T2D, since the reduction of BWV, among other risk factors and mechanisms, [29] may help to minimize the cardiovascular risk of such population.

## Limitations of the study

Our study has strengths and limitations. Strengths are: the large sample size of patients with T2D, the population-based design, minimizing selection bias, the inclusion of people free of cardiovascular complications at the entry and the follow-up duration of median 4.4 years. Limitations are related to the impossibility of establishing whether the correlation between BWV and cardiovascular complications is effectively causal and of identifying a possible mechanism for such causal correlation, albeit the design of the study, i.e. calculating BWV until a selected cut-off point and then evaluating its effects on the development of events beyond that period, might sustain the argument of causality. On the other hand, BWV might have changed during the observation phase, possibly affecting classification. In addition, albeit we adjusted for all the risk factors commonly used to estimate CV risk in clinical practice, we did not have data relative to dietary habits and physical activity of the patients, which are increasingly emerging as key drivers of CV complications, also irrespectively of body weight [30, 31]. Finally, we cannot establish if BWV was ascribable to intentional, *e.g.* dieting or introduction of additional glucose-lowering drugs, or involuntary factors, *e.g.* the development of a disease promoting cachexia such as kidney disease or cancer, a variable that might have influenced the results.

## Conclusion

Our finding shows that BWV may dramatically impact the development of cardiovascular complications in T2D suggests that any strategy to reduce the body weight in these patients should be aimed at maintaining the reduction in the long-term, avoiding oscillations.

## Supplementary Information

**Additional file 1: Table S1. **Codes of International Classification of Diseases, 9th Revision and 10th Revision for the outcomes assessed. **Table S2.** Crude number of events and event rate (events per 100 patient-years) according to quartiles of body weight variability for all the outcomes assessed.

## Data Availability

The datasets generated and/or analysed during the current study are not publicly available due to the Swedish legislation, but are available from the corresponding author on reasonable request.

## Abbreviations

95% CI: 95% Confidence interval
BWV: Body weight variability
eGFR: Estimated glomerular filtration rate
HRs: Hazard ratios
HDL: High-density lipoprotein cholesterol
LDL: Low-density lipoprotein cholesterol
NDR: Swedish National Diabetes Register
T2D: Type 2 diabetes

## References

[1] Lissner L, Odell PM, D'Agostino RB (1991). Variability of body weight and health outcomes in the Framingham population. N Engl J Med.

[2] Choi D, Choi S, Park SM (2019). Impact of weight variability on mortality among Korean men and women: a population based study. Sci Rep.

[3] Kim MK, Han K, Park YM, Sang H (2018). Associations of variability in blood pressure, glucose and cholesterol concentrations, and body mass index with mortality and cardiovascular outcomes in the general population. Circulation.

[4] Bangalore S, Fayyad R, Laskey R, DeMicco DA, Messerli FH, Waters DD (2017). Body-weight fluctuations and outcomes in coronary disease. N Engl J Med.

[5] Rawshani A, Rawshani A, Franzén S (2017). Mortality and cardiovascular disease in Type 1 and Type 2 Diabetes. N Engl J Med.

[6] Bangalore S, Fayyad R, DeMicco DA, Colhoun HM, Waters DD (2018). Body weight variability and cardiovascular outcomes in patients with type 2 diabetes mellitus. Circ Cardiovasc Qual Outcomes.

[7] Yeboah P, Hsu FC, Bertoni AG, Yeboah J (2019). Body Mass Index, change in weight, body weight variability and outcomes in type 2 diabetes mellitus (from the ACCORD Trial). Am J Cardiol.

[8] Doehner W, Erdmann E, Cairns R, Clark AL, Dormandy JA, Ferrannini E, Anker SD (2012). Inverse relation of body weight and weight change with mortality and morbidity in patients with type 2 diabetes and cardiovascular co-morbidity: an analysis of the PROactive study population. Int J Cardiol.

[9] Nam GE, Kim W, Han K (2020). Body weight variability and the risk of cardiovascular outcomes and mortality in patients with type 2 diabetes: a nationwide cohort study. Diabetes Care.

[10] Tancredi M, Rosengren A, Svensson AM (2015). Excess mortality among persons with Type 2 Diabetes. N Engl J Med.

[11] Ceriello A, Prattichizzo F (2021). Variability of risk factors and diabetes complications. Cardiovasc Diabetol.

[12] Zoppini G, Verlato G, Targher G, Bonora E, Trombetta M, Muggeo M (2008). Variability of body weight, pulse pressure and glycaemia strongly predict total mortality in elderly type 2 diabetic patients. The Verona Diabetes Study. Diabetes Metab Res Rev.

[13] Strohacker K, Carpenter KC, McFarlin BK (2009). Consequences of weight cycling: an increase in disease risk?. Int J Exerc Sci.

[14] Turicchi J, O'Driscoll R, Horgan G (2020). Body weight variability is not associated with changes in risk factors for cardiometabolic disease. Int J Cardiol Hypertens..

[15] Strohacker K, McFarlin BK (2010). Influence of obesity, physical inactivity, and weight cycling on chronic inflammation. Front Biosci (Elite Ed).

[16] Li X, Jiang L, Yang M, Wu YW, Sun JZ (2018). Impact of weight cycling on CTRP3 expression, adipose tissue inflammation and insulin sensitivity in C57BL/6J mice. Exp Ther Med.

[17] Yatsuya H, Tamakoshi K, Yoshida T (2003). Association between weight fluctuation and fasting insulin concentration in Japanese men. Int J Obes Relat Metab Disord.

[18] Ceriello A, Motz E (2004). Is oxidative stress the pathogenic mechanism underlying insulin resistance, diabetes, and cardiovascular disease? The common soil hypothesis revisited. Arterioscler Thromb Vasc Biol.

[19] de Candia P, Prattichizzo F, Garavelli S (2019). Type 2 diabetes: how much of an autoimmune disease?. Front Endocrinol (Lausanne).

[20] Prattichizzo F, Giuliani A, Sabbatinelli J (2020). Prevalence of residual inflammatory risk and associated clinical variables in patients with type 2 diabetes. Diabetes Obes Metab.

[21] La Sala L, Prattichizzo F, Ceriello A (2019). The link between diabetes and atherosclerosis. Eur J Prev Cardiol.

[22] Sarin HV, Lee JH, Jauhiainen M (2019). Substantial fat mass loss reduces low-grade inflammation and induces positive alteration in cardiometabolic factors in normal-weight individuals. Sci Rep.

[23] Piening BD, Zhou W, Contrepois K (2018). Integrative personal omics profiles during periods of weight gain and loss. Cell Syst.

[24] American Diabetes Association (2021). Obesity management for the treatment of type 2 diabetes: standards of medical care in diabetes—2021. Diabetes Care.

[25] Wing RR, Lang W, Wadden TA (2011). Look AHEAD Research Group. Benefits of modest weight loss in improving cardiovascular risk factors in overweight and obese individuals with type 2 diabetes. Diabetes Care.

[26] Wing RR, Bolin P, Brancati FL (2013). Look AHEAD Research Group. Cardiovascular effects of intensive lifestyle intervention in type 2 diabetes. N Engl J Med.

[27] Look AHEAD Research Group; Gregg E, Jakicic J, Blackburn G, et al. Association of the magnitude of weight loss and changes in physical fitness with long-term cardiovascular disease outcomes in overweight or obese people with type 2 diabetes: a post-hoc analysis of the Look AHEAD randomised clinical trial. Lancet Diabetes Endocrinol. 2016;4:913–21. 10.1016/S2213-8587(16)30162-0 PMC5094846 27595918

[28] Hill AJ (2004). Does dieting make you fat?. Br J Nutr.

[29] Prattichizzo F, La Sala L, Rydén L (2019). Glucose-lowering therapies in patients with type 2 diabetes and cardiovascular diseases. Eur J Prev Cardiol.

[30] Jenkins DJA, Dehghan M, Mente A (2021). Glycemic index, glycemic load, and cardiovascular disease and mortality. N Engl J Med.

[31] Rennemark M, Jogréus C, Elmståhl S, Welmer A, Wimo A, Sanmartin-Berglund J (2018). Relationships between frequency of moderate physical activity and longevity: an 11-year follow-up study. Gerontol Geriatr Med.

