# Supplementary material for: Variability in body weight and the risk of cardiovascular complications in type 2 diabetes: results from the Swedish National Diabetes Register
Source: Cardiovasc Diabetol. 2021 Aug 26;20:173. doi: 10.1186/s12933-021-01360-0 (PMC8394543; doi:10.1186/s12933-021-01360-0)
Supplement: Supplementary file 1 — Additional file 1: Table S1. Codes of International Classification of Diseases, 9th Revision and 10th Revision for the outcomes assessed. Table S2. Crude number of events and event rate (events per 100 patient-years) according to quartiles of body weight variability for all the outcomes assessed. [file 12933_2021_1360_MOESM1_ESM.docx]

**Additional appendix for**

**Variability in body weight and the risk of cardiovascular complications in type 2 diabetes. Results from the Swedish National Diabetes Register**

**Table S1.** Codes of International Classification of Diseases, 9th Revision and 10th Revision for the outcomes assessed.

**
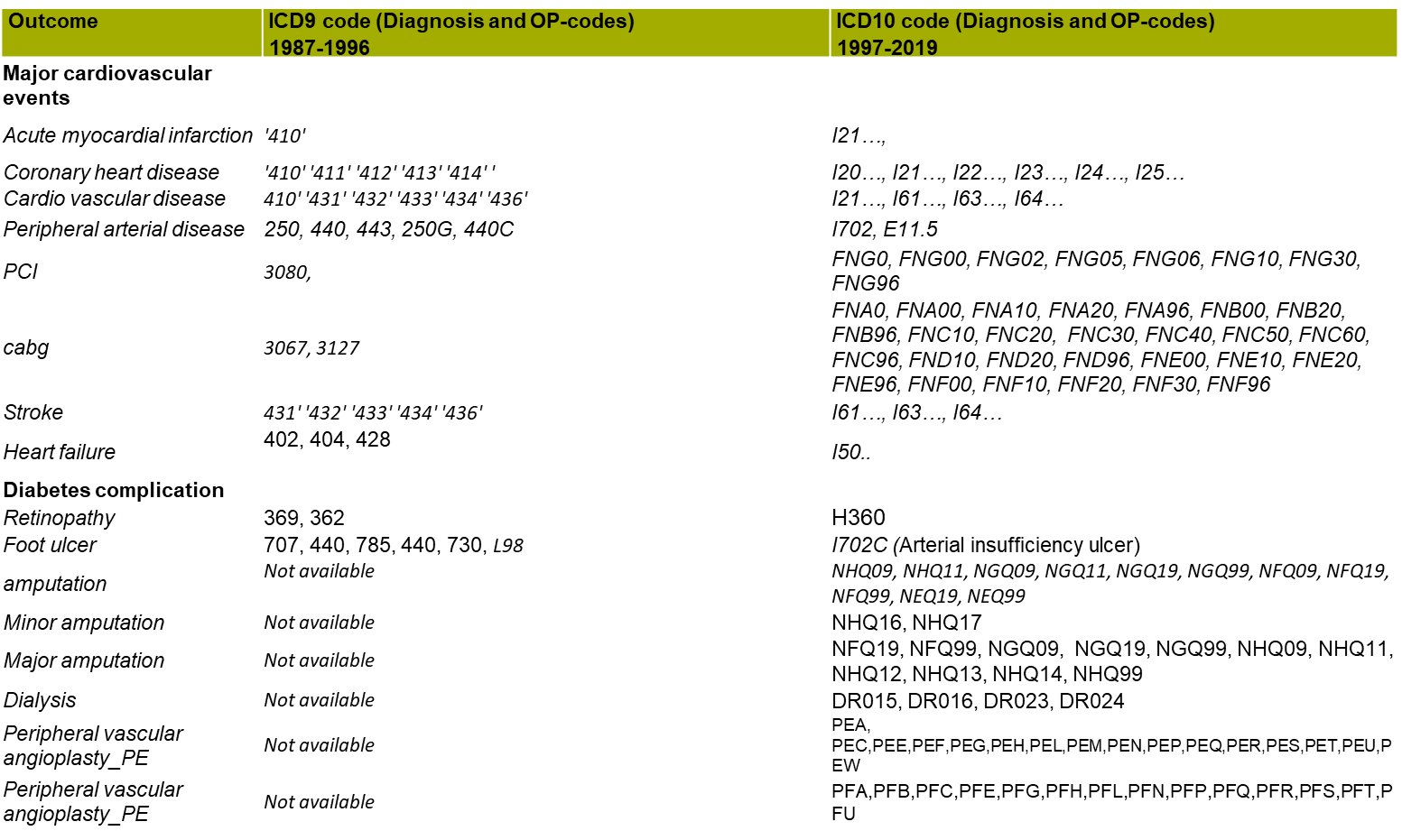
**

**Table S2.** Crude number of events and event rate (events per 100 patient-years) according to quartiles of body weight variability for all the outcomes assessed.

| Outcome | | Quartile I | Quartile II | Quartile III | Quartile IV |
| --- | --- | --- | --- | --- | --- |
|  |  | # events (events per 100  patient-years) | # events (events per 100  patient-years) | # events (events per 100  patient-years) | # events (events per 100  patient-years) |
| Primary composite outcome** | | 3997 (3.27) | 3896 (3.24) | 3662 (3.21) | 3670 (3.50) |
| Expanded composite outcome*** | | 4409 (3.65) | 4256 (3.58) | 3991 (3.54) | 3970 (3.82) |
| All-cause mortality | | 2794 (2.17) | 2749 (2.17) | 2659 (2.22) | 2759 (2.51) |
| Myocardial infarction | | 967 (0.78) | 874 (0.71) | 787 (0.68) | 700 (0.66) |
| Stroke | | 1004 (0.81) | 937 (0.77) | 816 (0.70) | 757 (0.71) |
| PCI | | 756 (0.61) | 701 (0.57) | 622 (0.54) | 543 (0.51) |
| CABG | | 281 (0.22) | 233 (0.19) | 201 (0.17) | 178 (0.17) |
| Hospitalization for heart failure | | 1386 (1.11) | 1312 (1.07) | 1315 (1.14) | 1340 (1.26) |
| Peripheral artery disease | | 356 (0.28) | 319 (0.26) | 304 (0.26) | 346 (0.32) |
| Lower limb revascularization (PE/PF) | | 310 (0.25) | 252 (0.20) | 246 (0.21) | 348 (0.23) |
| Foot ulcer | | 165 (0.13) | 156 (0.13) | 172 (0.15) | 188 (0.17) |
|  | * Baseline covariates included in the model: age, gender, duration of diabetes, body weight, smoking, HbA1c, systolic and diastolic blood pressure, total cholesterol, HDL, LDL, triglycerides, albuminuria, eGFR, retinopathy, treatment for diabetes, hypertension, dyslipidemia, and aspirin.  ** Non-fatal myocardial infarction, non-fatal stroke, all-cause mortality  *** Non-fatal myocardial infarction, non-fatal stroke, all-cause mortality, coronary revascularization/reperfusion (PCI, CABG) procedures, peripheral revascularization procedures. | | | | |
